# The Small Hole for the Kitten

**Published:** 1917-04

**Authors:** 


### The Small Hole for the Kitten.

There is a story of a Greek sophist who cut a hole in the lower part of his door to accommodate his cat and, beside it, a smaller hole so that the kitten could also go in and out at will. Somewhat the same principle is suggested in various ingenious medical propositions, e.g. to administer a thread soaked in congo red and partly inclosed in a capsule containing a "heavy powder giving a neutral reaction." After fifteen minutes, the thread is withdrawn, being colored dark blue or violet according to the amount of HCl in the stomach. If it is still red, "this shows anacidity (sic) or that the capsule has stuck somewhere on the way." There are various other factors that may produce a negative reaction when there is really HCl present, as the dilution or possibly neutralization by mucus in the oesophagus, and saliva. Congo red is not a thoroughly satisfactory indicator of inorganic acids but, even if dimethyl-amido azo-benzol were substituted —this being the best direct indicator for free HCl thus far discovered—the test would still be fallacious unless strongly positive and, even then, we should be in the position of the curious man who asked how a stranger had lost his leg. The latter promised to tell him if the former would promise not to ask any more questions and then gave the explanation that his leg had been bitten off. We would still be curious to know how much HCl was present.

In our experience, patients object to threads, and sounds as much as to the stomach tube. Why not cut one hole in the door for the cat, even if it is a little larger than that needed for the kitten?

